# Cricket (*Gryllus bimaculatus*) meal pellets as a protein supplement to improve feed efficiency, ruminal fermentation and microbial protein synthesis in Thai native beef cattle

**DOI:** 10.5713/ab.23.0107

**Published:** 2023-06-26

**Authors:** Burarat Phesatcha, Kampanat Phesatcha, Maharach Matra, Metha Wanapat

**Affiliations:** 1 Department of Applied Biology, Faculty of Sciences and Liberal Arts, Rajamangala University of Technology Isan, Nakhon Ratchasima 30000, Thailand; 2 Department of Animal Science, Faculty of Agriculture and Technology, Nakhon Phanom University, Nakhon Phanom 48000, Thailand; 3 Tropical Feed Resources Research and Development Center (TROFREC), Department of Animal Science, Faculty of Agriculture, Khon Kaen University, Khon Kaen 40002, Thailand

**Keywords:** Digestibility, *Gryllus bimaculatus*, Protein Source, Rumen Fermentation, Ruminants

## Abstract

**Objective:**

Replacing soybean meal (SBM) with cricket (*Gryllus bimaculatus*) meal pellets (CMP) in concentrate diets was investigated for feed efficiency, ruminal fermentation and microbial protein synthesis in Thai native beef cattle.

**Methods:**

Four male beef cattle were randomly assigned to treatments using a 4×4 Latin square design with four levels of SBM replaced by CMP at 0%, 33%, 67%, and 100% in concentrate diets.

**Results:**

Results revealed that replacement of SBM with CMP did not affect dry matter (DM) consumption, while digestibilities of crude protein, acid detergent fiber and neutral detergent fiber were significantly enhanced (p<0.05) but did not alter digestibility of DM and organic matter. Increasing levels of CMP up to 100% in concentrate diets increased ruminal ammoniacal nitrogen (NH_3_-N) concentrations, blood urea nitrogen, total volatile fatty acids and propionate concentration (p<0.05), whereas production of methane and protozoal populations decreased (p<0.05). Efficiency of microbial nitrogen protein synthesis increased when SBM was replaced with CMP.

**Conclusion:**

Substitution of SBM with CMP in the feed concentrate mixture at up to 100% resulted in enhanced nutrient digestibility and rumen fermentation efficiency, with increased volatile fatty acids production, especially propionate and microbial protein synthesis, while decreasing protozoal populations and mitigating rumen methane production in Thai native beef cattle fed a rice straw-based diet.

## INTRODUCTION

The recent large human population increase impacts global food production systems which are already struggling to satisfy rising levels of consumer demand. The United Nations estimates the global population to be 9 billion people by 2050, with demand for meat and milk increasing by 58% and 70% respectively [1]. Livestock, particularly ruminants, are one of the primary and most important sources of animal products but cost of feed impacts the continued expansion of livestock production. Intensive livestock production systems rely heavily on soybean meal (SBM) as the most important source of protein and essential amino acids [2]. Novel approaches and alternative sustainable components to supplant conventional sources of protein in animal diets will be required in the future to satisfy the growing demand for animal food products, with insects showing promise as potential alternatives sources of plant proteins routinely supplied to animals [3]. There is growing interest in feeding insect products to pets, pigs, chickens and fish as well as other types of animals. However, few scientific studies have evaluated the use of insect products in ruminant diets [4,5]. No statistics on the commercial rearing of insects are currently available but many countries now farm insects such as crickets for the feed market, with annual production of insect meal expected to rise to 1.2 million tons by 2025 [6]. Several different types of insects including crickets, mealworms, grasshoppers, locusts, house fly maggots and black soldier fly (BSF) larvae have been investigated for potential use as domestic animal feed instead of SBM and tested for their fermentation profile, digestibility and methane production in the rumen [7]. Insects have high protein content (44% to 70%) and can be used to produce inexpensive protein for animals. The cricket (*Gryllus bimaculatus*) consists of 68.50% crude protein (CP) and 12.50% ether extract (EE) [8], with a wide variety of essential amino acids such as methionine, lysine, histidine, valine and leucine [7]. Insects are rich in a variety of nutrients including the vitamins niacin, vitamin B12, thiamine and riboflavin and minerals such as calcium, iron, magnesium, potassium, zinc and phosphorus [9]. Wang et al [10] reported that crickets have high CP content, with more than 55% of their dry matter (DM) composed of protein and 8.7% chitin, contributing to higher nutritional value than BSF larvae and mealworms [7]. Chitin is a long-chain polymer of N-acetylglucosamine, a nitrogenous chemical found in insects, that is only partially digested by the digestive tracts of animals [11]. Chung et al [12] reported that feed supplementation with chitin or chitosan improved interaction with bacterial cell walls, with the subsequent permeability alterations leading to decrease in the bacterial population. Many people are now considering insects as a source of protein in their diets because they are a more environmentally friendly option [3]. Pig [13], poultry [14], and fish [15] feeds now use insect-based protein in place of fishmeal or SBM. Ahmed et al [16] reported that *Acheta domesticus*, *Gryllus bimaculatus*, *Bombyx mori*, and *Brachytrupes portentosus* improved *in vitro* fermentation and maintained degradability when added to concentrate containing 25% SBM as supplements. Phesatcha et al [8] studied *in vitro* fermentation levels of SBM replacement with cricket meal at 100:0, 75:25, 50:50, 25:75, and 0:100 in concentrate diets. Results showed that ruminal fermentation improved, while protozoal population and methane production both decreased. No negative effects were recorded on growth, weight increase or feed intake when field cricket (*Teleogryllus mitratus*) was given as a partial replacement for SBM in the diet of 8 to 20-day-old broilers [10]. Permatahati et al [17] replaced fish meal with cricket meal in feed, leading to an increase in egg production of layer quails and improved egg quality, while up to 30% cricket meal used as a replacement for SBM had no negative impacts on rumen fermentation profiles in post-weaning goat kids [18]. However, limited studies exist on the impact of cricket meal pellets (CMP) partially replacing SBM on nutrient digestibility, rumen fermentation and methane emission. Therefore, this study investigated how the replacement of SBM with CMP in concentrate diets impacted feed efficiency, ruminal fermentation and microbial protein synthesis in Thai native beef cattle.

## MATERIALS AND METHODS

### Ethical procedure

This study was approved by the Animal Care and Use Committee, Rajamangala University of Technology Isan, Thailand (approval no. 4/2565), with approval also required from the Thailand National Research Council’s Ethics of Animal Experimentation to collect rumen fluid from animals. The main study objective involved the laboratory examination of ruminant feeds.

### Preparation of cricket meal pellets

Adult field crickets (*Gryllus bimaculatus*) about 45 days old were sourced from a cricket farm in Khon Kaen Province. The heads, wings, and legs were removed before drying at 60 °C in a hot air oven. Cricket meal was processed by grinding the dried bodies to 1 mm (Cyclotech Mill; Foss Tecator AB, Höganäs, Sweden) and mixing 90% dried cricket powder, 9% cassava chips and 1% molasses. Cricket meal pellets were produced using a pellet machine (Victor pellet mill, China) and then sun-dried to achieve 90% DM before feeding to the animals.

### Dietary treatments and experimental design

Four Thai native male beef cattle (about two years old) with starting body weight (BW) 230±15 kg were randomly assigned treatments using a 4×4 Latin square design, with four treatment levels of SBM replacement with CMP at 0%, 33%, 67%, and 100% in concentrate diets. All the beef cattle were fed concentrate supplemented at 0.5% BW, with untreated rice straw chopped to 3 to 5 cm fed *ad libitum* at 07.00 and 16.00 hours. The dietary treatments were applied to the animals over the course of four separate periods, each lasting for 21 days. The animals had unrestricted access to water, with mineral lick blocks provided twice a day. Mineral lick block contained Cu 150 mg/kg, Co 25 mg/kg, I 50 mg/kg, Mn 500 mg/kg, Se 15 mg/kg, Zn 500 mg/kg and Na 328 mg/kg. Intake measurements were conducted throughout the first 14 days, with collection of total urine and feces continued during the last 7 days. Table 1 presents the ingredient proportions in the CMP concentrate mixtures and rice straw.

### Sample collection and chemical analyses

Feed amounts of roughage, and concentrate were identified daily by weighing the feed that was offered and the feed that was left uneaten after the morning feeding and combined by period for chemical analysis. Feed, feces, and urine samples were collected for analysis during the last 7 days of each period. After oven-drying, composite samples were ground (1 mm screen using a Cyclotech Mill; Foss Tecator AB, 1093, Sweden) and then analyzed for DM, ash, CP, EE [19], and acid insoluble ash (AIA). An internal indigestible flow marker known as AIA was used to calculate the digestibility of nutrients [20]. According to Van Soest et al [21], they were examined for acid detergent fiber (ADF) and neutral detergent fiber (NDF) of the nutrients. On the last day of each cycle, rumen fluid and blood samples were taken immediately after the morning feeding and again four hours later. Approximately 200 mL of fluid was collected from the ventral sac of the rumen using a stomach tube connected to a vacuum pump. The temperature and pH values (Hanna Instrument HI8424 Microcomputer, San Francisco, CA, USA) of the rumen fluid samples were immediately measured and recorded as the first stage of the process. The fluid samples obtained from the rumen were then passed through four layers of cheesecloth and separated into two equal parts. The first part was used to analyze ammonia nitrogen (NH_3_-N) and volatile fatty acids (VFA), which involved adding 5 mL of 1 M H_2_SO_4_ to 45 mL of rumen fluid before centrifuging the mixture at 1,600× g for 15 minutes. The sample was analyzed using high performance liquid chromatography (HPLC) (Model Water 600; UV detector, Millipore Crop, Cortland, NY, USA) for VFA [22] and NH_3_-N using the micro-Kjeldahl technique [19]. The second part was subjected to the total direct counting approach for bacteria, protozoa, and fungi populations using a haemocytometer according to the method of Galyean [23]. Rumen methane (CH_4_) production was approximated according to Moss et al [24] using VFA proportions, which are as follows: production of CH_4_ = 0.45 (acetate, C_2_)+0.275 (propionate, C_3_) +0.4 (butyrate, C_4_). Blood samples of 10 milliliters were taken from a jugular vein, stored in tubes containing ethylenediaminetetraacetic acid, and then utilized for blood urea nitrogen (BUN) according to the method of Crocker [25]. Allantoin and creatinine concentrations in urine were analyzed by HPLC, based on the Chen and Gomes [22], and the quantity of microbial purines ingested was determined from purine derivative excretion. The microbial crude protein (MCP) (g/d) at 3.99 ×0.856×mmol of purine derivatives excreted was determined following the method of Galo et al [26].

### Statistical analysis

The general linear model in SAS software [27] was used to examine data variances using a 4×4 Latin square design. Treatment trends were statistically compared using orthogonal polynomial contrasts. Differences among treatment means with p<0.05 as representing statistically significant differences.

## RESULTS AND DISCUSSION

### Chemical composition of experimental feeds

Table 1 shows the nutrient contents of commonly used diets. All the different concentrates used had 16.0% CP content. As CMP replaced SBM, dietary CP values for diet formulation were similar across diets. In this study coconut meal contained 18.0% CP, SBM contained 44% CP and cricket meal contained 67.0% CP. The CMP had high concentrations of 62.4% CP with 6.9% crude fiber and 14.7% EE, while rice straw (2.4 CP, 75.2% NDF and 46.1% ADF) was fed as a roughage source. Phesatcha et al [8] found that cricket meal contained CP and EE at 68.5% and 12.5%, respectively while cricket meal contained 67.7% CP, 14.5% EE, 10.8% ADF and 39.3% NDF according to Jayanegara et al [7]. Taufek et al [28] compared the chemical compositions of fish meal and cricket meal, with CP levels at 53.61% and 57.02%, respectively.

### Feed intake, growth performance and nutrient digestibilities

Changes in feed intake growth performance and nutrient digestibility when SBM was replaced with CMP are shown in Table 2. Results indicated that SBM substituted with CMP enhanced final BW and ADG (p<0.05). The treatments had no effect on total feed intake and rice straw amounts were similar (p>0.05). Consumption of mineral blocks was similar among treatments (p>0.05). Nutrient digestibilities of CP were significantly highest in the group with CMP replacing SBM at 100% (p<0.05), while digestibility of NDF and ADF decreased linearly when replacing SBM with CMP at 100% DM (p<0.05). Interestingly, CMP produced low digestibility due to high CF and EE contents which impacted the microorganisms responsible for food degradation in the rumen. DM consumption was not affected by the level of CMP in the concentrate mixture because CMP addition had no adverse impact on diet palatability. Addition of CMP with high protein, fat and essential amino acids improved ruminant feeding. Proteins derived from insects can supplement or even completely substitute conventional feed sources due to their high quality and diverse amino acid profiles [29]. Van Huis [30] demonstrated the potential of using insect protein in livestock feeds including BSF, cricket, housefly, silkworm, grasshopper, yellow mealworm and house fly maggots. Astuti and Komalasari [31] found that substituting SBM with cricket diet up to 30% did not affect rumen framing patterns or daily weight gain, while Phesatcha et al [32] found that supplementation of *Mitragyna speciosa* Korth leaf pellets improved DM intake and apparent digestibility in Thai native beef cattle by impacting microbial growth. The extruded pellets demonstrated good quality and palatability. Increasing the level of CMP to replace SBM in the concentrate mixture greatly improved nutrient digestibility but had no effect on the digestibility of DM and organic matter (OM). Chitin, as the primary component of cricket exoskeletons, is high in fiber and encourages the development of certain types of bacteria that have beneficial effects on intestinal health and the immune system [33]. Chitin is found in crustaceans and insects. Insects have high fiber content due to the existence of chitin and high protein and fat content. Phesatcha et al [8] explained that cumulative gas production at 96 h and *in vitro* DM degradability both increased with increasing levels of CM replacing SBM. In an *in vitro* digestibility experiment, Jayanegara et al [7] found that insect meals including *Tenebrio molitor*, *Gryllus assimilis*, and *Hermetia illucens* had lower DM and OM digestibility than SBM. They reported that high fiber contents in feeds containing insects reduced the digestibility of both DM and OM in comparison to SBM. They also reported that insect feeds typically contain 40% more DM than Jamaican field cricket, with higher fiber content than SBM, comparable with our findings. In terms of *in vitro* rumen fermentation, insect feed such as BSF larvae gave substantially higher levels of NDF and ADF. Fukuda et al [34] evaluated the effects of BSF larvae as a protein supplement in cattle steers. They discovered that animals receiving insect meals showed higher weight gain, without any adverse effects on digestibility.

### Rumen fermentation characteristics, blood metabolites and microbial populations

When SBM was replaced with CMP, ruminal temperature and pH were not statistically different among treatments (p>0.05), as shown in Table 3. Results showed that when SBM was substituted with CMP, ruminal pH ranged from 6.7 to 6.8. When increasing the level of CMP in the concentrate as replacement for SBM, concentrations of ammoniacal nitrogen (NH_3_-N) and BUN increased significantly (p<0.05). The concentration of NH_3_-N in the rumen increased from 13.6 to 18.4 mg/dL when increasing the levels of CMP replacing SBM because of increased protein digestion in CMP (62.5% CP) and the concentrate mixture (16.0% CP). Diets rich in CP stimulated the growth of microbes in the rumen, particularly proteolytic bacteria which are responsible for breaking down protein and deaminating it into NH_3_-N [35]. In the rumen, this nitrogen supply is used as a precursor for the production of amino acids and proteins which are then synthesized by microbes [36]. This result concurred with Jayanegara et al [37], who showed that NH_3_-N formation in the rumen depended on the total amount of protein present and on a number of other significant factors including protein fraction and the rate of protein degradation. Hristov and Ropp [38] suggested that the inability of microorganisms to use NH_3_-N for microbial protein synthesis may be linked to the absence of highly degradable carbohydrates. BUN concentration significantly improved when replacing SBM with CMP, ranging from 12.5 to 13.7 mg/dL, while concentrations of urea nitrogen in the blood corresponded with the availability of ammonia nitrogen in the rumen. Elevated BUN concentration levels are often associated with increased protein degradation in the rumen and could be used to identify nitrogen metabolism in ruminants because increased microbial protein synthesis reduces the rumen ammonia nitrogen concentration.

Total VFA and propionate (C_3_) also markedly increased, whereas acetate (C_2_) and the C_2_:C_3_ ratio significantly reduced (p<0.05). The concentration of butyrate was not affected (p>0.05) by CMP replacing SBM, while methane concentration linearly (p<0.05) reduced with CMP replacing SBM at 100% in the diet. When increasing the level of CMP as replacement for SBM in concentrate diets, the bacterial population increased while the protozoal population significantly decreased (p<0.05), with no change in fungal zoospore counts (p>0.05). Increasing the level of CMP in feed reduced rumen methane production and protozoal numbers because insects have high fat content. Schauff et al [39] reported a decrease in total VFA and acetate with increase in propionate, resulting in decreased acetate to propionate ratio when supplementing tallow for whole soybean. From a nutritional point of view, the consumption of fat by ruminants, regardless of the type of fat consumed, results in reduced methane production. Insect oils high in 12:0 and 14:0 also have a beneficial influence on ruminal function by inhibiting the production of methane and ammonia respectively [40]. Methane emissions reduce when EE levels are elevated because certain fatty acids, particularly medium chain fatty acids (MCFA), are toxic to methanogens [41]. Dietary fat influences fiber particles in the rumen by covering the surface of fiber cells, thereby preventing degradation by microbes. MCFA directly inhibit rumen methanogenic archaea and may change their metabolic activity, as well as the composition of the rumen methanogenic population. Increase in bacterial population enhances rumen fermentation, resulting in greater total VFA and propionate production. Russell et al [42] stated the positive association of ruminal microorganism numbers to feed digestibility, which plays a significant and vital role in feed-substrate degradation, particularly with bacteria and fungi. However, some results suggested that chitosan (a chitin deacetylation derivative) decreased methane production by modifying bacterial populations [43,44]. Phesatcha et al [8] found that replacing SBM with CM improved total VFA and propionate proportions but decreased acetate proportions and methane production in an *in vitro* experiment.

### Microbial protein synthesis

Urinary PD, allantoin absorption and excretion were similar among treatments (p>0.05), while microbial protein supply and efficiency of microbial nitrogen synthesis (EMNS) considerably improved (p<0.05) when replacing SBM with CMP at 100% in the diet (Table 4). Nitrogen absorption and retention were both useful predictors of how effectively the ruminants utilized the nutrients [22]. Interestingly, the values of excreted allantoin were closer to those registered for buffalo cows than for Fresian cows [45]. Highest levels of CMP resulted in the most efficient and abundant microbial protein synthesis. Urinary purine derivative excretion and absorption did not vary significantly between treatments, whereas microbial nitrogen synthesis (MNS) and EMNS significantly improved (p<0.05) when SBM was replaced with CMP. The results of this study showed that CMP were affected by CP only at very high concentrations by rendering more protein available for digestion in the lower gut which eventually increased microbial protein. Therefore, higher CMP replacement was more effective in microbial protein synthesis. The protein composition of insects must also be taken into consideration. Firkins et al [46] explained that most proteins provided to the small intestine of ruminants could be supplied by microbial protein synthesis that occurs in the rumen at between 50% and 80% of the overall absorbable protein. Supplementation of *Flemingia macrophylla* pellets at 150 g per day in lactating dairy cows greatly improved nitrogen absorption and retention [47]. Phesatcha et al [48] also demonstrated that MCP and EMNS, comprising dietary protein and CT, were the outcome of high CP in the concentrate diet and MPLP-SBM supplementation.

## CONCLUSION

Substitution of SBM with CMP in the feed concentrate mixture at up to 100% resulted in enhanced nutrient digestibility and rumen fermentation efficiency, with increased VFA production, especially propionate and microbial protein synthesis, while decreasing protozoal populations and mitigating rumen methane production in Thai native beef cattle fed a rice straw-based diet.

## Figures and Tables

**Table 1:** Feed ingredients, concentrate mixtures and chemical compositions

Items	Replacement levels of CMP for SBM (% fresh basis)				CMP	RS

	0	33	67	100		
Ingredients (%, fresh basis)						
Cassava chip	54.0	58.0	62.0	62.0	2	
Soybean meal	12.0	8.0	4.0	0.0		
Cricket meal pellet	0.0	4.0	8.0	12.0	95	
Rice bran	3.0	5.5	8.6	9.5		
Coconut meal	19.0	11.5	6.5	5.0		
Palm kernel meal	5.4	6.4	4.4	5.0		
Molasses	2.0	2.0	2.0	2.0	1	
Urea	1.6	1.6	1.5	1.5		
Sulfur	1.0	1.0	1.0	1.0	1	
Salt	1.0	1.0	1.0	1.0		
Mineral premix	1.0	1.0	1.0	1.0	1	
Chemical composition (% of dry matter)						
Dry matter (%)	92.1	91.5	94.7	95.2	96.6	89.7
Ash	6.3	6.8	7.1	6.5	4.1	10.3
Crude protein	16.4	16.1	16.3	16.3	62.4	2.4
Neutral detergent fiber	23.4	22.6	20.9	20.1	-	75.2
Acid detergent fiber	20.1	17.6	16.5	17.2	-	46.1
Crude fiber	-	-	-	-	6.9	-
Ether extract	2.5	7.1	8.5	12.5	14.7	-

CMP, cricket meal pellet; RS, rice straw.

**Table 2:** Cricket meal pellet as a protein source on voluntary feed intake and nutrient digestibility in beef cattle

Items	Replacement levels of CMP for SBM (% fresh basis)				SEM	Contrasts		
	
	0	33	67	100		Linear	Quadratic	Cubic
Initial BW (kg)	212	210	210	214	0.97	0.58	0.12	1.00
Final BW (kg)	241^c^	253^b^	260^a^	263^a^	2.49	0.02	0.77	0.30
Average daily gain (kg/d)	0.43^b^	0.46^b^	0.54^a^	0.58^a^	0.93	0.01	0.17	0.47
Rice straw intake								
Kg of DM/d	2.5	2.5	2.7	2.8	0.10	0.06	0.88	0.53
% of BW	1.2	1.2	1.3	1.3	0.05	0.06	0.90	0.54
g/kg BW^0.75^	45.0	45.8	49.5	50.3	1.89	0.06	1.05	0.53
Concentrate intake								
Kg of DM/d	1.0	1.1	1.1	1.3	0.12	0.17	0.32	0.60
% of BW	0.5	0.5	0.5	0.5	-	-	-	-
g/kg BW^0.75^	19.0	19.3	19.1	23.7	2.03	0.17	0.33	0.59
Mineral block intake								
Kg of DM/d	0.05	0.04	0.04	0.04	0.31	0.20	0.41	0.50
Total feed intake								
Kg of DM/d	3.5	3.6	3.8	4.1	0.19	0.06	0.48	0.97
% of BW	1.7	1.7	2.8	1.8	0.05	0.06	0.90	0.54
g/kg BW^0.75^	64.0	65.1	68.6	74.0	3.33	0.06	0.54	0.97
Apparent digestibility (%)								
Dry matter	60.5	62.6	67.8	65.9	3.56	0.22	0.59	0.53
Organic matter	63.0	67.5	61.3	64.0	6.29	0.08	0.92	0.90
Crude protein	64.3^b^	65.3^b^	69.1^a^	68.2^a^	3.63	<0.05	0.60	0.31
Neutral detergent fiber	49.5^a^	49.3^a^	48.7^a^	46.1^b^	5.53	<0.05	0.35	0.63
Acid detergent fiber	35.4^a^	34.8^a^	35.1^a^	31.7^b^	3.97	<0.05	0.51	0.30

CMP, cricket meal pellet; SBM, soybean meal; SEM, standard error of the means; BW, body weight; DM, dry matter.

a–c Means in the same row with different superscripts differed (p<0.05).

**Table 3:** Cricket meal pellet as a protein source on fermentation characteristics, blood urea nitrogen and microbial population in beef cattle

Items	Replacement levels of CMP for SBM (% fresh basis)				SEM	Contrasts		
	
	0	33	67	100		Linear	Quadratic	Cubic
Ruminal pH	6.77	6.94	6.81	6.98	0.11	0.28	0.66	0.58
Temperature (°C)	38.0	38.2	38.8	38.6	0.18	0.61	0.53	0.94
Ammonia-nitrogen (mg %)	13.6^a^	16.1^ab^	17.2^b^	18.4^c^	0.96	<0.05	0.60	0.40
Blood urea nitrogen (mg/dL)	12.5^a^	12.8^a^	13.3^b^	13.7^b^	0.14	<0.05	0.55	0.56
Total VFAs (mg/dL)	86.5^a^	100.7^ab^	103.4^ab^	109.5^b^	4.77	<0.05	0.43	0.51
Molar of VFAs (%)								
Acetic acid (C_2_)	71.5^a^	68.0^ab^	63.8^ab^	61.2^b^	2.36	<0.05	0.86	0.83
Propionic acid (C_3_)	21.9^b^	23.8^a^	25.3^a^	26.1^a^	2.25	<0.05	0.80	0.86
Butyric acid (C_4_)	12.3	11.6	10.8	10.3	1.89	0.40	0.76	0.70
C_2_/C_3_ ratio	3.3^a^	2.9^ab^	2.5^ab^	2.3^b^	0.24	<0.05	0.77	0.99
CH_4_ estimation (mmol/100 mol)^1)^	31.0^a^	28.7^ab^	25.7^b^	24.5^b^	1.20	<0.05	0.64	0.66
Rumen microbe population (cell/mL)								
Bacteria (×10^11^)	4.6^a^	5.4^ab^	6.3^bc^	6.5^c^	0.93	<0.05	0.94	0.95
Protozoa (×10^6^)	6.4^a^	5.1^ab^	5.3^ab^	4.1^b^	1.02	<0.05	0.72	0.18
Fungi (×10^5^)	2.4	2.6	2.5	2.5	0.62	0.25	0.74	0.77

CMP, cricket meal pellet; SBM, soybean meal; SEM, standard error of the means; VFAs, volatile fatty acids

1) CH_4_, Methane production = 0.45 (C_2_)–0.275 (C_3_)+0.4 (C_4_) calculated according to Moss et al [24].

a–c Means in the same row with different superscripts differed (p<0.05).

**Table 4:** Cricket meal pellet as a protein source on urinary purine derivatives (PD) and microbial protein synthesis in beef cattle

Items	Replacement levels of CMP for SBM (% fresh basis)				SEM	Contrasts		
	
	0	33	67	100		Linear	Quadratic	Cubic
Urinary purine derivatives (mmol/d)								
Allantoin excretion	29.7	28.5	25.6	25.8	2.42	0.21	0.79	0.68
Allantoin absorption	77.6	85.6	89.3	90.7	2.34	0.79	0.50	0.98
MNS (g/d)	56.5^a^	61.2^b^	64.8^bc^	65.7^c^	4.18	<0.05	0.74	0.99
EMNS (g/kg OMDR)	24.8^a^	28.9^a^	30.7^a^	31.4^b^	2.54	<0.05	0.09	0.77

CMP, cricket meal pellet; SBM, soybean meal; SEM, standard error of the mean; MNS, efficiency of nitrogen synthesis; EMNS, efficiency of microbial nitrogen synthesis; OMDR, digestible organic matter apparently fermented in the rumen.

a–c Means in the same row with different superscripts differed (p<0.05).
